# Hidden and Under-Recognized Causes of Sudden Unexpected Death in Infancy (SUDI): A Comprehensive Review of Autopsy Findings

**DOI:** 10.3390/diagnostics16111730

**Published:** 2026-06-04

**Authors:** Jessika Camatti, Anna Laura Santunione, Rossana Cecchi, Erjon Radheshi, Edoardo Carretto, Maria Paola Bonasoni

**Affiliations:** 1 Department of Medicine and Surgery, University of Parma, Via Università 12, 43121 Parma, Italy; jessika.camatti@unipr.it; 2 Department of Biomedical, Metabolic and Neural Sciences, Unit of Legal Medicine, University of Modena and Reggio Emilia, Via Campi 287, 41125 Modena, Italy; annalaura.santunione@unimore.it (A.L.S.); rossana.cecchi@unimore.it (R.C.); 3 Unit of Legal Medicine and Bioethics, Azienda USL-IRCCS di Reggio Emilia, Via Amendola 2, 42122 Reggio Emilia, Italy; erjon.radheshi@ausl.re.it; 4 Local Health Authority ‘Papa Giovanni XXIII’, 24127 Bergamo, Italy; edoardo.carretto@asst-pg23.it; 5 Department of Medical and Surgical Sciences, Unit of Legal Medicine, University of Bologna, Via Irnerio 49, 40126 Bologna, Italy; 6 Pathology Unit, Azienda USL-IRCCS di Reggio Emilia, Via Amendola 2, 42122 Reggio Emilia, Italy

**Keywords:** sudden unexpected death in infancy (SUDI), sudden infant death syndrome (SIDS), post-mortem diagnostics, multidisciplinary investigation, medicolegal assessment

## Abstract

Sudden unexpected death in infancy (SUDI) remains a major challenge in pediatric pathology and forensic medicine. Despite advances in diagnostic techniques, many cases are still classified as unexplained and labeled as sudden infant death syndrome (SIDS). Increasing evidence suggests that a proportion of these deaths may be due to “hidden” causes not detectable through routine post-mortem examination. A narrative review of the literature (2000–2026) was conducted using PubMed and Scopus, focusing on under-recognized causes of SUDI and their diagnostic implications. Relevant studies were selected and organized into major pathological and forensic categories. Hidden causes of SUDI include a wide spectrum of conditions. Cardiac disorders—such as myocarditis, cardiomyopathies, and inherited arrhythmogenic syndromes—are frequently implicated and may require molecular autopsy for detection. Infectious diseases, often presenting with minimal or nonspecific findings, represent another important category, particularly viral and bacterial infections. Inborn errors of metabolism, especially fatty acid oxidation defects, may lead to sudden death in the absence of specific autopsy findings, highlighting the role of biochemical analyses. Neuropathological abnormalities involving brainstem regulatory systems may contribute to impaired autonomic control. Environmental, toxicological, and medico-legal factors—including unsafe sleep conditions, toxic exposures, and inflicted injury—must also be considered. SUDI is a multifactorial entity in which many unexplained deaths may be attributable to identifiable but overlooked conditions. A standardized, multidisciplinary approach integrating autopsy, ancillary investigations, and molecular diagnostics is essential to improve diagnostic accuracy and support prevention strategies.

## 1. Introduction

Sudden unexpected death in infancy (SUDI) remains one of the most challenging entities in pediatric pathology and forensic medicine [1,2]. According to the CDC, SUDI includes three major categories: SIDS, deaths of unknown cause, and Accidental Suffocation and Strangulation in Bed (ASSB) [3]. This classification highlights the heterogeneity of sudden infant deaths and reinforces the importance of standardized autopsy protocols and death scene investigation in differentiating explained from unexplained cases. Despite advances in preventive strategies and diagnostic techniques, a substantial proportion of SUDI cases continues to be labeled as unexplained, reflecting persistent limitations in post-mortem evaluation and diagnostic interpretation [2,4].

To date, significant variability persists in autopsy practices, diagnostic criteria, and classification systems across institutions and countries, contributing to inconsistencies in cause-of-death determination and epidemiological surveillance [2,5].

In recent years, increasing attention has been directed toward so-called “hidden” causes of SUDI—conditions that may not be evident on routine macroscopic examination and require targeted histological, microbiological, toxicological, biochemical, or molecular analyses for detection [4,6,7,8,9]. In addition, environmental, external, and medico-legal factors—including unsafe sleep conditions and, in rare cases, inflicted injury—may interact with intrinsic vulnerabilities, further complicating the interpretation and classification of findings. The growing availability of advanced diagnostic techniques, particularly molecular autopsy and genetic testing, has also expanded the spectrum of detectable causes in previously unexplained deaths [6,9].

This comprehensive review aims to provide an integrated and diagnostically oriented overview of the main hidden causes of SUDI, with particular emphasis on autopsy findings and post-mortem investigative strategies. Rather than offering a quantitative synthesis, the purpose of this review is to critically examine the spectrum of under-recognized conditions. The focus is specifically placed on diagnostically challenging causes of SUDI that may remain undetected during routine post-mortem examination. More common causes of infant mortality, including prematurity-related complications, low birth weight, malnutrition, congenital anomalies, and healthcare-related disparities, were considered beyond the specific scope of the present review.

## 2. Materials and Methods

This study was designed as a comprehensive narrative review aimed at providing an integrated diagnostic and pathological overview of hidden causes of SUDI.

A literature search was conducted using PubMed (MEDLINE) and Scopus databases to identify relevant publications addressing SUDI, autopsy findings, and under-recognized causes of death. The search strategy combined Medical Subject Headings (MeSH) and free-text terms, including “sudden unexpected death in infancy”, “SUDI”, “SIDS”, “post-mortem”, “autopsy”, “molecular autopsy”, and “cause of death”, along with additional terms related to cardiac, infectious, metabolic, neurological, environmental, toxicological, and forensic conditions.

The literature search was last updated in April 2026. The search primarily focused on articles published in English between 2000 and 2026. Additional relevant studies were identified through manual screening of reference lists and citation tracking to ensure broad and comprehensive coverage of the topic. Eligible sources included original studies, autopsy-based case series, clinical-pathological investigations, consensus statements, and narrative or systematic reviews considered relevant to the diagnostic and forensic evaluation of SUDI. Studies were selected based on their relevance to the diagnostic and forensic evaluation of SUDI, with particular emphasis on those addressing pathological findings, ancillary investigations (including histology, microbiology, toxicology, and molecular analyses), and diagnostic challenges in post-mortem practice. Studies not directly related to post-mortem evaluation, forensic investigation, or hidden/under-recognized causes of SUDI were excluded from the narrative synthesis.

Given the comprehensive and narrative nature of this review, no formal systematic review methodology or meta-analysis was applied. The selection of studies was guided by the authors’ judgment of scientific relevance and contribution to the understanding of hidden causes of SUDI.

The retrieved literature was analyzed and organized into thematic categories according to major diagnostic domains, including cardiac, infectious, metabolic, neurological, environmental, toxicological, and medico-legal causes. Findings were synthesized narratively with the aim of providing a clinically and forensically oriented overview, highlighting key diagnostic features, potential pitfalls, and the role of multidisciplinary investigation. This approach was intended to provide an integrative perspective on the complexity of SUDI rather than a quantitative assessment of outcomes.

## 3. Diagnostic Approach and Autopsy Investigation in SUDI

A comprehensive and standardized diagnostic approach is essential in the evaluation of SUDI, as incomplete or inconsistent investigations may lead to misclassification and under-recognition of identifiable causes of death. SIDS remains a diagnosis of exclusion, requiring a thorough post-mortem examination, review of clinical history, and evaluation of the circumstances of death [10,11].

One of the major challenges in SUDI is the lack of uniformity in autopsy practices and diagnostic criteria. Significant variability persists among institutions and countries, affecting both the extent of investigations performed and the interpretation of findings. This heterogeneity contributes to inconsistencies in cause-of-death certification and hampers accurate epidemiological surveillance [12,13]. Furthermore, differences in classification systems and diagnostic preferences have led to a progressive shift from SIDS to broader or indeterminate categories, complicating comparisons across studies [14].

The importance of a complete and systematic autopsy cannot be overstated. Pediatric autopsy represents a complex and highly specialized procedure that must be tailored to the specific characteristics of infant deaths, where pathological findings may be subtle or minimal [15]. In this context, a “complete” autopsy extends beyond gross examination to include extensive histological sampling, toxicological screening, microbiological investigations, and molecular analyses [16,17]. A detailed examination of the brainstem autonomic nuclei and the cardiac conduction system through serial histological sections may reveal subtle developmental abnormalities and microscopic alterations not detectable by routine autopsy techniques.

Particular attention should be paid to the position in which the infant was found, sleep surface characteristics, bedding materials, co-sleeping arrangements, environmental conditions, timeline reconstruction, and photographic documentation of the death scene.

Despite these recommendations, autopsy practices remain inconsistent worldwide. The absence of universally adopted protocols contributes to diagnostic uncertainty and limits the reproducibility of findings. Standardized investigation systems have been proposed to address these issues, emphasizing the need for harmonization in both autopsy procedures and classification criteria [18].

The implementation of structured post-mortem protocols has been shown to significantly improve diagnostic accuracy in SUDI. In particular, the adoption of comprehensive autopsy approaches integrating histological, toxicological, and molecular analyses has led to a substantial reduction in the proportion of cases classified as unexplained, with a corresponding increase in the identification of infectious and inflammatory causes of death [19]. These findings support the concept that a significant fraction of SIDS cases may represent previously unrecognized pathological conditions.

In addition to the autopsy itself, DSI plays a critical role in the diagnostic process. The evaluation of environmental factors, sleeping position, and potential external contributors is essential to distinguish between natural causes, accidental asphyxia, and unexplained deaths [13,20].

Recent research has also highlighted the overlap between different categories of sudden death, including SIDS, SADS, and SUDEP, suggesting shared underlying mechanisms involving cardiac, neurological, and genetic factors [11,21].

Advances in forensic pathology have further emphasized the importance of integrating novel diagnostic tools into post-mortem investigations. Techniques such as molecular testing, immunohistochemistry, and advanced imaging have improved the detection of otherwise occult conditions and expanded the diagnostic spectrum of SUDI [17,22].

## 4. Under-Recognized Causes and Contributing Factors in SUDI

A wide spectrum of under-recognized pathological conditions, environmental contributors, and medico-legal factors may contribute to SUDI, many of which are not detectable through routine post-mortem examination. The conditions discussed below include both occult intrinsic pathological entities and environmental or circumstantial contributors that may complicate the diagnostic evaluation and classification of SUDI.

These “hidden” causes reflect the multifactorial nature of SUDI and often require targeted ancillary investigations. Importantly, the failure to recognize such conditions may lead to misclassification as SIDS, with significant implications for epidemiology, family counseling, and prevention strategies.

### 4.1. Cardiac Causes

Cardiac disorders represent one of the most relevant and diagnostically challenging categories of hidden causes in SUDI. These conditions often remain undetected during routine post-mortem examination, particularly in the absence of overt macroscopic abnormalities, and require a comprehensive approach integrating histological, molecular, and genetic investigations [23,24,25].

Structural and inflammatory cardiac diseases constitute an important subset of identifiable causes. Myocarditis has emerged as a significant contributor to sudden infant death, although its diagnosis remains complex and requires strict histopathological criteria to avoid overinterpretation [26]. Previous autopsy-based studies have shown that cardiac alterations, including myocarditis and hypoxia-related changes, are relatively frequent findings in SUDI cases, although their causal role must be carefully evaluated [27].

In addition to inflammatory conditions, cardiomyopathies represent another important group of hidden cardiac causes. These may include early or subclinical forms of hypertrophic, dilated, or arrhythmogenic cardiomyopathies, which may not be evident on gross examination and may require detailed histological and molecular evaluation for diagnosis. Expert cardiac examination is therefore essential, as both underdiagnosis and overinterpretation of subtle findings may lead to misclassification, with significant implications for family counseling and risk assessment [23].

A particularly important and increasingly recognized category is represented by primary electrical disorders, or channelopathies. These include long QT syndrome, Brugada syndrome, and other inherited arrhythmogenic conditions that can lead to sudden death in structurally normal hearts. Molecular autopsy studies have demonstrated that a proportion of SIDS cases harbor pathogenic or potentially significant variants in genes associated with cardiac ion channels, supporting a genetic predisposition to lethal arrhythmias [24]. Similarly, earlier investigations have suggested that mutations in ion channel-related genes may be present in a substantial subset of SIDS cases, reinforcing the role of genetic screening in unexplained deaths [28]. However, the interpretation of post-mortem genetic findings remains challenging, particularly in the presence of variants of uncertain significance, incomplete penetrance, or limited genotype–phenotype correlation. Therefore, molecular findings should always be interpreted within a multidisciplinary clinical, pathological, and forensic context.

Long QT syndrome has been identified as one of the most frequent channelopathies associated with SUDI, with reported prevalence ranging widely across studies. Importantly, this condition is potentially preventable if identified early, highlighting the clinical relevance of post-mortem diagnosis and family screening [29]. The broader spectrum of arrhythmogenic disorders also includes other channelopathies and genetic conditions affecting cardiac excitability, which may remain entirely undetectable at conventional autopsy [30].

In this context, the study of the cardiac conduction system has gained increasing attention. Subtle developmental abnormalities or acquired lesions affecting the conduction pathways may predispose to lethal arrhythmias but are frequently overlooked in routine examinations. Advanced histopathological techniques, including serial sectioning of the conduction system, have demonstrated a range of structural alterations—such as hypoplasia, dispersion, and accessory pathways—that may contribute to sudden death in infants [25,31].

The need for standardized and detailed cardiac examination in cases of sudden death has been further reinforced by recent consensus recommendations. These guidelines highlight the importance of systematic macroscopic and microscopic evaluation, appropriate tissue sampling, and the integration of ancillary investigations, including genetic testing, to improve diagnostic accuracy and identify heritable conditions [32].

### 4.2. Infectious Causes

Infectious diseases represent one of the most significant and potentially under-recognized categories of hidden causes of SUDI. In many cases, infections may be subtle, paucisymptomatic, or entirely clinically silent, and autopsy findings can be minimal or nonspecific, thereby complicating accurate cause-of-death determination and contributing to potential misclassification within the SUDI spectrum [33,34].

Respiratory infections are among the most frequently implicated conditions in SUDI identified at autopsy. Mild or even subclinical infections of the upper or lower respiratory tract may act as triggering factors through mechanisms such as hypoxia, apnea, or dysregulation of autonomic control, particularly in vulnerable infants [35].

Central nervous system infections, including meningitis and encephalitis, also represent important hidden causes that may be missed during standard post-mortem evaluation. In infants, these conditions may present with minimal inflammatory response, especially in early stages or in the setting of an immature immune system. Molecular post-mortem studies have demonstrated the presence of infectious agents in cases initially classified as unexplained, supporting the role of infection as a contributing or triggering factor [34].

Sepsis and systemic infections constitute another critical category in forensic evaluation. Bacterial pathogens, particularly *Escherichia coli* and *Staphylococcus aureus*, have been implicated in SUDI, often in association with toxin-mediated mechanisms and abnormal host inflammatory responses [36,37]. However, the interpretation of microbiological findings remains challenging, as post-mortem bacterial translocation and contamination may confound results. Therefore, microbiological data must always be interpreted in conjunction with histopathological findings and clinical context to avoid both overdiagnosis and underdiagnosis [38]. Similarly, the detection of microbial DNA or RNA through molecular techniques does not necessarily establish causality, particularly in the absence of supporting histopathological or clinical evidence.

Myocarditis of infectious origin represents a relevant overlap between infectious and cardiac causes of SUDI. Viral myocarditis, most commonly associated with enteroviruses, may present with focal and subtle myocardial inflammation that can be easily missed without targeted sampling [36,37].

Viral pathogens implicated in SUDI during post-mortem investigation extend beyond cardiotropic viruses and include adenovirus, influenza virus, respiratory syncytial virus, cytomegalovirus, Epstein–Barr virus, and parvovirus B19. These agents may contribute to death through multiple mechanisms, including direct tissue injury, systemic inflammatory responses, and interference with autonomic and respiratory regulation [35,39]. Notably, viral genomes may be detectable in multiple organs even in the absence of overt histological changes, highlighting the importance of molecular diagnostics in identifying otherwise hidden causes [35,40].

A major diagnostic challenge in infectious SUDI lies in distinguishing true causative infections from incidental findings or post-mortem artifacts. The mere presence of microorganisms is insufficient to establish causality; instead, a multidisciplinary interpretation integrating histopathology, microbiology, and clinical data is required [34,38]. In this context, the lack of standardized protocols for microbiological sampling and interpretation represents a significant limitation in current practice [40,41].

Advances in molecular pathology, including PCR and NGS, have significantly enhanced the detection of infectious agents in post-mortem tissues. These techniques enable the identification of viral and bacterial DNA or RNA even in cases with minimal or absent inflammatory response. However, their increased sensitivity also raises concerns regarding specificity and clinical relevance [38,39]. Indeed, misclassification of infection-related deaths as SIDS has important implications for epidemiology, prevention strategies, and family counseling [42].

### 4.3. Metabolic Causes

Metabolic disorders represent a well-recognized but often underdiagnosed category of hidden causes of SUDI. IEMs, particularly those affecting energy production and substrate utilization, may lead to sudden death in previously healthy infants, frequently in the absence of specific macroscopic findings at autopsy [43,44].

Although relatively rare, metabolic diseases are estimated to account for a small but significant proportion of SUDI cases, ranging from approximately 0.9% to 6%, depending on the population studied and the extent of post-mortem investigation [44]. Importantly, many of these conditions are potentially identifiable through targeted biochemical and molecular analyses [43].

Disorders of FAO represent the most frequently implicated metabolic conditions in SUDI. These defects impair mitochondrial β-oxidation, leading to energy deficiency, hypoketotic hypoglycemia, and accumulation of toxic metabolites, particularly during periods of fasting or intercurrent illness. MCADD is among the most common FAO disorders and has been consistently associated with sudden and unexpected infant death [45]. The clinical presentation may be nonspecific or entirely absent prior to death.

Severe and early-onset FAO disorders, such as MTPD and LCHADD, may present with rapid metabolic decompensation and sudden death in the neonatal period. Post-mortem findings often include diffuse hepatic steatosis and lipid accumulation in cardiac muscle, reflecting the underlying defect in energy metabolism [46]. These conditions may manifest before newborn screening results become available, further complicating early diagnosis [44].

Other FAO-related disorders, including CACTD, are characterized by severe metabolic crises with hypoglycemia, hyperammonemia, and cardiac dysfunction, often leading to death within the first days of life. Autopsy findings may reveal vacuolar degeneration in the liver and myocardium, while genetic analysis is required to confirm the diagnosis [47].

Beyond FAO disorders, a wide spectrum of IEMs has been associated with SUDI, including organic acidemias, urea cycle disorders, mitochondrial diseases, and disorders of amino acid metabolism. Although less frequent, these conditions may present with acute metabolic decompensation or sudden collapse, sometimes without clear preceding symptoms. Rare entities such as L-2-hydroxyglutaric aciduria have also been reported in association with sudden infant death [44,48].

A major diagnostic challenge in metabolic SUDI lies in the frequent absence of specific gross pathological findings. Consequently, the diagnosis relies heavily on biochemical analyses, including acylcarnitine profiling, amino acid analysis, and detection of metabolic intermediates in blood, urine, or bile [43].

Advances in laboratory techniques, particularly tandem mass spectrometry, have significantly improved the post-mortem detection of metabolic disorders. These methods allow for the identification of characteristic metabolic signatures even in small or degraded samples, thereby increasing diagnostic yield [43].

However, the accuracy of these investigations depends on the timely collection and proper preservation of biological specimens.

NBS programs have substantially reduced mortality associated with several metabolic diseases by enabling early diagnosis and treatment. Nevertheless, some infants may die before screening results are available or in regions where screening panels are limited, underscoring the continued relevance of post-mortem metabolic investigations [44,46]. Indeed, FAO disorders alone are estimated to account for up to 5% of sudden infant deaths in certain cohorts [46].

Emerging evidence also suggests that metabolic derangements, such as metabolic acidosis and electrolyte imbalance, may contribute to the pathophysiology of sudden death, either as primary mechanisms or as secondary effects of underlying conditions. These alterations may interact with other factors, including infection and cardiac instability, further complicating the interpretation of findings [49].

### 4.4. Neurological Causes

A substantial body of evidence suggests that dysfunction of central autonomic and respiratory control mechanisms—especially at the level of the brainstem—may underlie a significant proportion of sudden infant deaths [50,51].

The brainstem plays a crucial role in the regulation of vital functions, including respiration, cardiovascular control, arousal, and responses to hypoxia and hypercapnia. Neuropathological and experimental studies have consistently demonstrated that abnormalities affecting these regulatory networks may impair the infant’s ability to respond to life-threatening challenges during sleep, such as asphyxia or hypoxia [50]. In particular, defects in autoresuscitation, altered respiratory patterns, and impaired arousal responses have been described in vulnerable infants. Detailed neuropathological examination of the brainstem by serial sections has identified developmental abnormalities involving nuclei responsible for autonomic and respiratory regulation in a subset of SUDI/SIDS cases [52,53].

One of the most influential models in this field is the serotonin brainstem hypothesis, which postulates that a subset of SIDS/SUDI cases is associated with abnormalities in serotonergic neurons within the medullary reticular formation [54]. These alterations may result in failure of protective reflexes during sleep, including arousal and ventilatory responses to hypoxia, thereby leading to sudden death. Neurochemical studies have further identified abnormalities in multiple neurotransmitter systems, including serotonin, acetylcholine, catecholamines, and substance P, supporting the concept of a broader dysfunction of the neurochemical network regulating cardiorespiratory control [55].

Beyond neurochemical alterations, structural and developmental abnormalities of the central nervous system have also been described. These include microdysgenesis of the brainstem, abnormalities of the hippocampus, and alterations in hypothalamic nuclei involved in autonomic regulation [50,56]. However, the interpretation of these findings remains challenging, as many changes are subtle and may overlap with normal anatomical variants.

Another key component in the pathophysiology of neurologic SUDI is the dysfunction of central and peripheral chemoreceptors, which are essential for detecting changes in oxygen and carbon dioxide levels. Alterations in these systems may impair ventilatory responses and arousal mechanisms, contributing to fatal outcomes under hypoxic conditions [57].

The neuronal respiratory network has also been implicated in sudden infant death. Disruptions in the maturation or function of brainstem respiratory circuits may lead to instability in breathing patterns and failure of compensatory mechanisms during sleep [51]. These findings further support the concept that SUDI may result from an interaction between intrinsic vulnerability and external stressors.

In addition to brainstem-related mechanisms, epilepsy represents an important overlapping domain. SUDEP shares clinical, pathological, and genetic features with SUDI, including the involvement of ion channel dysfunction and autonomic instability [57]. Molecular studies have demonstrated that variants affecting ion channel genes may predispose to both arrhythmias and seizures, suggesting a shared pathophysiological substrate between neurological and cardiac causes of sudden death [57].

### 4.5. Environmental and Circumstantial Contributors

Environmental and circumstantial contributors represent a critical and often under-recognized category of deaths within the SUDI spectrum, particularly in cases where external circumstances play a decisive role. Unlike many intrinsic causes, these conditions are not solely identifiable through autopsy findings and require careful integration of scene investigation, clinical history, and environmental assessment for accurate classification [20,58].

One of the major challenges in this field is the distinction between accidental asphyxia and SIDS. As emphasized by Matshes et al., apparent asphyxial deaths may present with minimal or nonspecific autopsy findings, making it difficult to establish causality in the absence of detailed contextual information [59]. This diagnostic overlap contributes to significant variability in cause-of-death certification and may lead to both under- and over-classification of asphyxial deaths [12].

The sleep environment plays a central role in many of these cases. Unsafe sleep conditions—including prone positioning, soft bedding, bed-sharing, and overlaying—have been consistently associated with an increased risk of death [1,58,60]. Infants who die in shared sleeping situations have been shown to differ from those who die while sleeping alone, with higher rates of potentially hazardous environmental factors [61]. Accurate reconstruction of the sleep environment through standardized DSI protocols is essential to differentiate accidental asphyxia, unsafe sleep-related deaths, and unexplained natural deaths.

From a pathophysiological perspective, several mechanisms have been proposed to explain asphyxia-related deaths in infancy. These include airway obstruction, external compression of the thorax, and rebreathing of carbon dioxide in confined or poorly ventilated environments. The anatomical characteristics of the upper airway in early infancy—such as relative narrowing and increased collapsibility—may further predispose infants to airway compromise under adverse conditions [62].

DSI is therefore essential in the evaluation of suspected asphyxial deaths. As demonstrated by Erck Lambert et al., variability in the quality and completeness of scene investigations significantly impacts diagnostic accuracy and classification [19]. Standardized approaches to DSI, including detailed documentation of sleep position, bedding, and environmental conditions, are therefore crucial.

### 4.6. Toxicological and Environmental Exposures

In most cases, toxicological findings do not constitute a primary cause of death but rather act as contributory or aggravating factors, often interacting with underlying vulnerabilities or adverse environmental conditions.

Exposure to tobacco smoke is one of the most consistently identified and clinically relevant risk factors. Both prenatal and postnatal exposure to environmental tobacco smoke have been strongly associated with an increased risk of SIDS, with nicotine recognized as a key neurotoxic agent affecting cardiorespiratory regulation and arousal mechanisms [63,64]. Nicotine readily crosses the placenta and accumulates in fetal tissues, where it may interfere with brainstem development and impair the infant’s response to hypoxic stress, a mechanism considered central in SUDI pathophysiology [63]. Epidemiological evidence further supports tobacco exposure as one of the leading preventable risk factors for sudden infant death [64].

Environmental pollutants also play a potential role. Exposure to air pollutants such as NO_2_ and particulate matter has been associated with increased infant mortality, including SIDS, although the strength of this association remains modest and subject to methodological limitations [65]. Case–control studies have additionally suggested a possible link between acute exposure to nitrogen dioxide and increased risk of SIDS, particularly in the days immediately preceding death [66].

Direct toxicological causes of death in infancy are rare but well documented. Fatal intoxications, including opioid exposure such as methadone, have been reported in isolated cases, highlighting the importance of considering accidental or non-accidental poisoning in forensic investigations [67]. However, interpretation of toxicological results in infants is particularly challenging due to age-related pharmacokinetic differences and the lack of established reference ranges.

Recent studies have emphasized the value of advanced toxicological techniques, including hair analysis, in identifying chronic or repeated exposure to drugs. In cohorts of SUDI cases, polysubstance exposure has been frequently detected and is often associated with multiple concurrent risk factors, suggesting that toxicological findings may reflect broader environmental and social vulnerabilities rather than isolated causative events [68].

A major limitation in this field is the difficulty in distinguishing causation from incidental exposure. The detection of toxic substances does not necessarily imply a direct role in death, and results must be interpreted in conjunction with autopsy findings, scene investigation, and clinical history. In many cases, toxicological factors contribute to a multifactorial risk model, consistent with the widely accepted “triple risk” hypothesis of SIDS.

### 4.7. Unnatural Causes: Child Abuse and Medico-Legal Considerations

Fatal maltreatment may clinically and pathologically mimic natural causes of death, particularly SIDS, making differentiation extremely challenging and heavily dependent on a comprehensive forensic investigation [69].

In many cases, abusive deaths present with nonspecific or absent external findings, especially in instances of smothering or covert homicide. The distinction between inflicted asphyxia and natural unexplained death may be impossible based solely on autopsy findings, requiring integration of scene investigation, clinical history, and multidisciplinary assessment [70]. Indeed, a proportion of deaths historically classified as SIDS may represent undetected homicides, although the exact prevalence remains debated and likely lower than earlier estimates.

Emergency and forensic studies have attempted to identify features suggestive of abuse in SUDI cases. Factors such as prior involvement of child protective services, the presence of sentinel injuries, and unexpected clinical findings (e.g., return of spontaneous circulation after resuscitation) have been associated with abusive deaths, although no single feature is sufficiently specific to establish causality [69].

A thorough post-mortem investigation is essential in suspected cases of fatal abuse. Standard forensic protocols emphasize the integration of complete autopsy, death scene investigation, caregiver interviews, and review of medical and social history [69]. Ancillary investigations play a crucial role: post-mortem radiography can identify occult skeletal injuries, including metaphyseal fractures and rib fractures, which may not be detected during routine autopsy and are highly suggestive of inflicted trauma [71]. Similarly, specialized examinations such as ocular autopsy can reveal retinal hemorrhages and optic nerve findings characteristic of abusive head trauma, contributing significantly to diagnostic accuracy [72].

The medico-legal implications of misclassification are substantial. Failure to recognize abusive deaths may result in missed opportunities for child protection and prevention of further harm, while overdiagnosis carries serious legal and ethical consequences. Therefore, current evidence strongly supports a cautious, multidisciplinary, and standardized approach to the investigation of SUDI, in which child abuse is systematically considered among the differential diagnoses.

Despite significant advances in post-mortem investigation, the diagnostic evaluation of SUDI remains affected by important limitations. Variability in autopsy practices, challenges in the interpretation of pathological and microbiological findings, and the increasing use of molecular analyses introduce potential sources of both underdiagnosis and overdiagnosis. In particular, distinguishing causative conditions from incidental findings—such as inflammatory infiltrates, microbial detection, or genetic variants of uncertain significance—remains a major challenge. These issues highlight the need for standardized protocols and a cautious, multidisciplinary approach to improve diagnostic accuracy and reduce misclassification. The main categories of hidden causes of SUDI, along with their associated diagnostic approaches and potential pitfalls, are summarized in Table 1.

## 5. Future Perspectives

Future research in SUDI is increasingly oriented toward the integration of advanced diagnostic technologies, particularly in the fields of molecular pathology, genomics, and digital health. Despite significant progress in post-mortem investigation, a substantial proportion of cases remains unexplained, highlighting the need for more refined and standardized approaches.

One of the most promising developments is the expanding role of molecular autopsy, which has emerged as a key tool in the investigation of unexplained infant deaths. Genetic analysis can identify pathogenic variants associated with cardiac channelopathies, metabolic disorders, and other inherited conditions that are not detectable through conventional autopsy alone [73,74]. Recent studies suggest that SUDI and SIDS represent heterogeneous entities with multifactorial and often genetically mediated mechanisms, reinforcing the importance of integrating genomic data into routine post-mortem protocols [74,75].

Advances in NGS, including whole-exome and whole-genome sequencing, have significantly improved the detection of rare and novel variants associated with sudden death in infancy. These technologies, when applied in the peri-mortem setting, offer the potential to increase diagnostic yield and identify previously unrecognized genetic conditions [76].

Beyond traditional pathology, emerging technologies such as artificial intelligence (AI) and digital health tools may play an increasing role in both research and prevention. AI-based systems, including large language models, have shown potential in providing accessible and reliable health information related to SIDS, although their clinical applicability requires further validation [77]. Similarly, digital health interventions—such as mobile applications, telemedicine, and online educational platforms—represent promising strategies for improving parental awareness and adherence to safe sleep practices [77].

## 6. Conclusions

This review highlights that a wide spectrum of “hidden” causes in SUDI cases—including cardiac, infectious, metabolic, neurological, environmental, and toxicological factors—may underlie deaths initially labeled as unexplained. Many of these conditions are characterized by subtle or absent macroscopic findings and can only be identified through a comprehensive and multidisciplinary approach integrating histology, microbiology, toxicology, and molecular analyses.

Among these causes, particular attention should be given to inherited cardiac conditions, occult infections, and inborn errors of metabolism, which not only contribute to mortality but also have important implications for family counseling and prevention of recurrence. Similarly, environmental and external factors—including sleep-related conditions and, in rare cases, abusive mechanisms—must be carefully evaluated through thorough DSI and standardized protocols.

The findings of this review reinforce the concept that SIDS should be considered a diagnosis of exclusion, achievable only after a complete and methodologically rigorous investigation. The integration of emerging tools such as molecular autopsy and genetic testing is progressively expanding the diagnostic spectrum, allowing the identification of previously unrecognized causes of death. Ancillary molecular and microbiological findings should always be interpreted within the broader forensic, pathological, and clinical context to minimize the risk of overinterpretation or misclassification.

Improving the accuracy of SUDI diagnosis requires a standardized, multidisciplinary, and highly specialized approach, combining traditional autopsy with advanced ancillary techniques. Such an approach is essential to reduce the proportion of unexplained cases, enhance epidemiological reliability, and provide meaningful information for clinical management, family counseling, and public health strategies aimed at preventing sudden infant deaths.

## Figures and Tables

**Table 1:** Main categories of hidden causes of SUDI and associated diagnostic approaches.

Category	Examples	Key Diagnostic Tools	Main Pitfalls
**Cardiac causes**	Myocarditis, cardiomyopathies, channelopathies (e.g., long QT syndrome, Brugada syndrome), histopathological anomalies of the cardiac conduction system	Histology, immunohistochemistry, molecular autopsy (genetic testing)	Absence of macroscopic findings; focal inflammation; variants of uncertain significance
**Infectious causes**	Viral infections (e.g., enterovirus, RSV), bacterial sepsis (e.g., *E. coli*, *S. aureus*), CNS infections	Microbiology, PCR, molecular assays, histology	Post-mortem contamination; minimal inflammatory response; difficulty in establishing causality
**Metabolic causes**	Fatty acid oxidation defects (e.g., MCADD), urea cycle disorders, mitochondrial diseases	Biochemical analyses (acylcarnitine profile), toxicology, genetic testing	Lack of specific autopsy findings; sample degradation; missed diagnosis without targeted testing
**Neurological causes**	Brainstem abnormalities, serotonergic dysfunction, epilepsy-related mechanisms	Neuropathology, immunohistochemistry, molecular studies	Subtle or nonspecific findings; overlap with normal variants; limited routine assessment
**Environmental/Asphyxial causes**	Unsafe sleep environment, prone position, bed-sharing, airway obstruction	Death scene investigation (DSI), clinical history correlation	Minimal autopsy findings; misclassification as SIDS; variability in scene investigation quality
**Toxicological causes**	Tobacco exposure, drug intoxication (e.g., opioids), environmental pollutants	Toxicological screening (blood, hair analysis), environmental assessment	Difficulty distinguishing causation vs incidental exposure; lack of reference ranges in infants
**Unnatural causes (abuse)**	Inflicted asphyxia, blunt trauma, covert homicide	Full forensic investigation, radiology, ocular examination	Absence of external injuries; overlap with natural causes; medico-legal implications

## Data Availability

No new data were created or analyzed in this study. Data sharing is not applicable to this article.

## Abbreviations

The following abbreviations are used in this manuscript:

LCHADD	Long-Chain 3-Hydroxyacyl-CoA Dehydrogenase Deficiency
MCADD	Medium-Chain Acyl-CoA Dehydrogenase Deficiency
SUDEP	Sudden Unexpected DEath in Epilepsy
CACTD	Carnitine-AcylCarnitine Translocase Deficiency
MTPD	Mitochondrial trifunctional protein deficiency
IEMs	Inborn errors of metabolism
SADS	Sudden Arrhythmic Death Syndrome
SUDI	Sudden Unexpected Death in Infancy
SIDS	Sudden Infant Death Syndrome
ASSB	Accidental Suffocation and Strangulation in Bed
CDC	Centers for Disease Control and Prevention
DSI	Death Scene Investigation
PCR	Polymerase Chain Reaction
FAO	Fatty Acid Oxidation
NBS	NewBorn Screening
NGS	Next-Generation Sequencing
NO_2_	Nitrogen diOxide
